# Risk factors for pulmonary hemorrhage of very low birth weight infants: a meta-analysis

**DOI:** 10.1186/s13052-025-02194-2

**Published:** 2026-02-03

**Authors:** Jie Wang, Jun Chen, Jipeng Shi, Yiheng Dai

**Affiliations:** Department of Neonatology, The Affiliated Foshan Women and Children Hospital, Guangdong Medical University, Foshan, 528000 China

**Keywords:** Pulmonary hemorrhage, Risk factor, Very low birth weight infants, Newborns, Meta analysis

## Abstract

**Background:**

Pulmonary hemorrhage (PH) is influenced by various perinatal factors. This study aims to identify the risk factors associated with pulmonary hemorrhage in very low birth weight infants and provide a reference for the prevention of this condition in this vulnerable population.

**Methods:**

A comprehensive literature search was conducted across several databases, including PubMed, Web of Science, Embase, Cochrane Library, Scopus, CNKI, CBM, Wanfang Database and VIP Database, covering publications up to May 20, 2024. The meta-analysis was performed using RevMan 5.4 and Stata 16.0 software to analyze the risk factors for pulmonary hemorrhage in very low birth weight infants.

**Results:**

A total of 14 studies were analyzed, comprising 13 case-control studies and 1 cohort study. The combined sample included 17,122 neonates, with 1,363 neonates in the case group and 15,759 neonates in the control group. The meta-analysis identified several risk factors for pulmonary hemorrhage in very low birth weight infants, including patent ductus arteriosus (OR = 2.95, 95% CI: 2.06–4.24), low 5-minute Apgar score (OR = 1.36, 95% CI: 1.01–1.84), use of surfactant (OR = 2.29, 95% CI: 1.40–3.75), respiratory distress syndrome (OR = 6.71, 95% CI: 3.67–12.28), and early-onset sepsis (OR = 6.02, 95% CI: 2.82–12.82). Protective factors identified included complete course of antenatal corticosteroids (OR = 0.36, 95% CI:0.17–0.79).

**Conclusions:**

Several risk factors, including patent ductus arteriosus, low 5-minute Apgar scores, use of surfactant, respiratory distress syndrome, and early-onset sepsis, are associated with an increased risk of pulmonary hemorrhage in very low birth weight infants. In contrast, complete course of antenatal corticosteroids has a protective effect in reducing the risk of this severe complication.

**Supplementary Information:**

The online version contains supplementary material available at 10.1186/s13052-025-02194-2.

## Introduction

In recent years, the incidence of very low birth weight infants (VLBWIs) has increased, largely due to rising pregnancy rates among older women and advances in assisted reproductive technologies. As a distinct subgroup, VLBWIs are particularly vulnerable to pulmonary hemorrhage (PH), owing to their reduced gestational age and low birth weight [1]. Very low birth weight (VLBW) is defined as a newborn with a birth weight of less than 1,500 g. Neonatal pulmonary hemorrhage is characterized by widespread bleeding in the terminal air sacs and pulmonary interstitium, often co-occurring with hyaline membrane disease [2, 3]. This condition is acute, catastrophic, and frequently life-threatening, leading to a rapid deterioration in the clinical status of neonates, especially VLBWIs. PH is typically recognized by the persistent discharge of fresh bloody fluid from the upper respiratory tract or through the endotracheal tube [4]. Histologically, it is defined by the presence of fresh blood within the alveolar spaces or pulmonary interstitium [5]. Pulmonary hemorrhage generally occurs within the first few days of life, with an incidence ranging from 1‰ to 12‰ [3]. It has a high mortality rate of approximately 50%. Previous studies have shown that PH incidence in VLBWIs ranges from 0.5% to 11%, depending on the criteria used to define both birth weight and pulmonary hemorrhage [6]. Infants with pulmonary hemorrhage often require prolonged respiratory support and are at higher risk for complications like bronchopulmonary dysplasia or chronic lung disease. The mortality rate is substantial, and even survivors face an increased risk of long-term issues such as cerebral palsy, cognitive impairment, seizures, and periventricular leukomalacia [7]. Thus, pulmonary hemorrhage poses a serious clinical challenge in VLBWIs.

The pathophysiology of neonatal pulmonary hemorrhage involves hemorrhagic edema, with severity ranging from mild, self-limiting cases to severe, progressively worsening syndromes that can be fatal [4]. Diagnosis is primarily based on clinical findings, including acute respiratory deterioration, blood-stained airway secretions and chest imaging showing diffuse pulmonary alveolar opacities, supplemented by laboratory markers of bleeding and inflammation [4, 8]. No definitive early diagnostic markers exist, making early detection reliant on clinicians’ vigilance. As a result, pulmonary hemorrhage is often underdiagnosed or misdiagnosed, with hemorrhagic fluid from the oro-nasal cavity or endotracheal tube observed in only about 50% of cases.

Due to these challenges in early detection and the severe clinical presentation, identifying risk factors for pulmonary hemorrhage is critical to improving prevention strategies, particularly in VLBWIs. While recent studies have explored potential risk factors [9–22] findings have varied across regions and studies. Systematic evaluation of these risk factors remains lacking, with most studies limited to single-center data. Multicenter studies, which provide broader case coverage and enhance generalizability, are needed to address this gap. To this end, a meta-analysis was conducted to systematically assess the risk factors for pulmonary hemorrhage in VLBWIs, aggregating data from multiple sources to inform better prevention and management strategies in clinical practice.

## Materials and methods

This study adhered to the guidelines outlined by the Preferred Reporting Items for Systematic Reviews and Meta-Analyses (PRISMA) [23]. Clinical trial number: not applicable.

### Literature search

A comprehensive search was conducted across multiple databases, including China National Knowledge Infrastructure (CNKI), China Biology Medicine Disc (CBM), Wanfang Database, VIP Database, Cochrane Library, PubMed, Web of Science, Excerpta Medica Database (Embase), and Scopus, as well as clinical trial registries such as the China Clinical Trial Registry and the US Clinical Trial Registry. The objective was to identify all studies reporting primary data on risk factors for pulmonary hemorrhage of VLBWIs. The search encompassed literature from the inception of each database up to May 20, 2024. The Chinese search was conducted using the terms “di chu sheng ti zhong er,” “di chu sheng ti zhong,” “di chu sheng zhi liang er,” “Fei chu xue,” and “Yin su” as subject terms or free words. Similarly, the English search employed the terms “Infant, Low Birth Weight,” “Pulmonary hemorrhage,” and “Factor(s)/risk” as subject terms or free words. Additionally, the literature from the included studies was reviewed, and their reference lists were manually searched to identify further eligible studies.

### Literature inclusion and exclusion criteria

Inclusion criteria: (1) Original studies on the risk factors of pulmonary hemorrhage in very low birth weight infants, utilizing data from the specified databases up to May 20, 2024; (2) Studies employing a case-control design or those in which the study population is divided into case and control groups, encompassing both cross-sectional and cohort that compare exposure factors between the two groups; (3) The diagnostic criteria of the disease and the definition and quantification of exposure factors are basically the same [4, 8]; (4) The studies directly or indirectly provides the OR (95% CI) of the exposure factors.(5) If multiple studies involving the same population have been published, preference was given to the study with the larger sample size or the most recent data.

Exclusion criteria: (1) Duplicate publications; (2) Reviews, systematic reviews, animal experiments; (3) Studies whose research content does not match the theme of this study; (4) Studies without rigorous experimental design; (5) Studies with inconsistent research subjects (the research subject is not very low birth weight infants); (6) Full text is unavailable; (7) Studies with inconsistent outcome indicators; (8) Conference papers or dissertations;

### Document data extraction

The literature was systematically screened, and data were independently extracted by two authors, followed by cross-verification. Any inconsistencies encountered were resolved through group discussion, with a third party making the final decision if necessary. Missing information was supplemented through direct contact with the original authors. Data sorting and summarization were conducted using Excel spreadsheets. The extracted data encompassed the name of the first author, year of publication, study area, research duration, study type, number of cases, number of controls, risk factors, odds ratio (OR), and 95% confidence interval (95% CI) for each variable evaluated as a potential predictor of pulmonary hemorrhage. Upon completion of the extraction process, a third party reviewed the extracted data to identify discrepancies. Any differences were resolved through group discussions and consultations with professional statisticians.

### Literature quality evaluation

Two authors independently assessed the quality of the literature and subsequently synthesized the findings. The quality evaluation of the case-control studies (*n* = 13) and the retrospective cohort study (*n* = 1) was conducted using the Newcastle-Ottawa Scale (NOS) [24]. Studies achieving a score of 7 or higher out of a possible 9 points were classified as high-quality literature.

### Strength of evidence

To determine the level of evidence for each influencing factor, existing evidence scales were employed for assessment, taking into account the quality of the studies. These scales were defined as follows [25]: (1) Strong evidence: the results come from a pool of three or more studies, at least two of which are high-quality homogeneous studies or synthesis of multiple high-quality studies (2) Moderate evidence: statistically significant results from a combination of one high-quality study and one or more studies of moderate or low quality (3) Limited evidence: the results come from a high-quality study or a combination of one or more moderate or low-quality studies, and (4) Very limited evidence: the no evidence: significantly pooled results from multiple studies where heterogeneity findings were unrelated to quality.

### Data synthesis

Upon reviewing and incorporating the relevant research literature, an evaluation of the employed statistical methods was conducted. The Odds Ratio (OR) was selected as the measure of effect size. The statistical significance was confirmed when *p* < 0.05. Results were presented as OR (95% CI). The data of all included risk factors (*n* = 14) were analyzed. Articles were grouped according to the type of risk factor using forest plots presenting results for the same factors. To ensure the reliability of the pooled effect estimates size, we only performed a meta-analysis of the risk factors assessed in at least three different studies. Risk factors derived from two or fewer studies were not subjected to meta-analysis.

### Statistical methods

Pooled odds ratios with corresponding 95% confidence intervals were calculated to estimate the effect of risk factors on the incidence of pulmonary hemorrhage. In cases where OR values were not available, software was employed for conversion. Heterogeneity was assessed using *I²* values and Cochrane’s Q statistic. An *I²* value greater than 50% or a *P*-value less than or equal to 0.1 indicated significant heterogeneity [26], analyzed using the random effects model (REM); otherwise, a fixed effects model (FEM) was utilized. By comparing the differences in the combined values of different effect models and one-by-one elimination method, the sensitivity of the research results is analyzed. Used funnel plots and Egger’s and Begg’s linear regression to assess potential publication bias. Funnel plot analysis was conducted only when at least ten studies were included in the analysis [23]. Forest plots were generated using Review Manager (RevMan) version 5.4 software, while the remaining statistical analyses, including sensitivity analysis and publication bias detection, were performed using Stata version 16.0 software.

## Results

### Literature screening results

A total of 1,399 documents were initially identified through the preliminary search, which was managed using NoteExpress software. The screening process was conducted in three stages. The search strategy was developed by the first two authors, with one author responsible for executing the search and screening the articles at each stage. To minimize bias during the selection and exclusion of studies, the second author reviewed the process at each level in accordance with Cochrane guidelines.

**Fig. 1:**
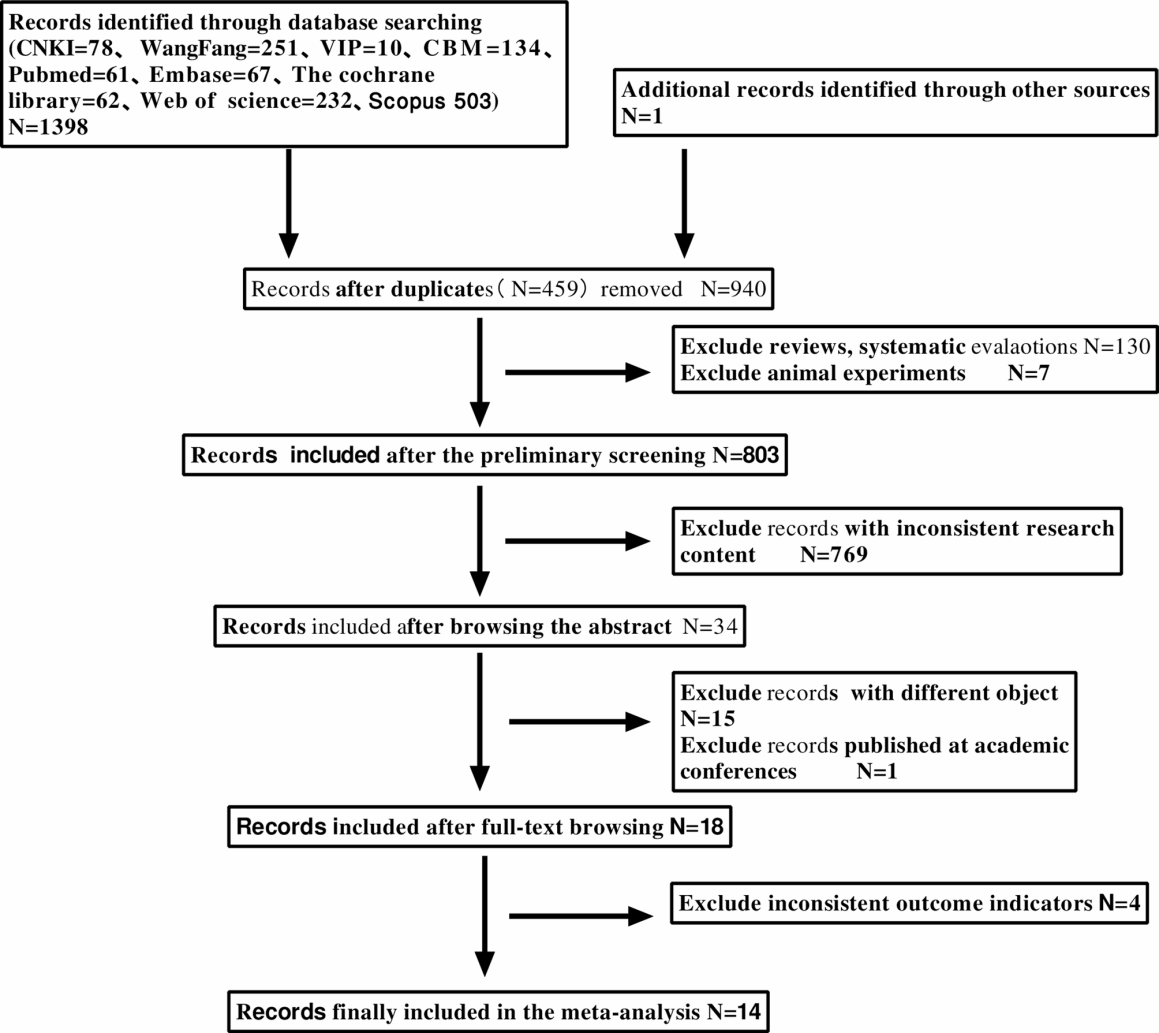
Document screening flow chart

Level 1: A total of 1,399 articles were evaluated based on their titles and abstracts. Articles were excluded if they were duplicates, reviews, systematic reviews, comments, or animal studies. After this step, 803 articles remained. Level 2: Screening further excluded articles that lacked sufficient information to meet the study’s inclusion criteria or were deemed irrelevant. This reduced the number of eligible studies significantly. Level 3: Data were extracted from 34 literature studies to obtain information. During this stage, some studies were found to have incomplete data, leading to attempts to contact the authors via email to request full publications. As a result, 15 papers were excluded due to inconsistent study populations, one was a conference paper, and four were excluded due to inconsistent outcome indicators. Two authors independently reviewed any potential conflicts during the screening process and reached a consensus on which studies to include, ultimately selecting 14 studies for the final analysis [9–22]. The literature screening process and outcomes are illustrated in Fig. 1.

### Characteristics of the included literature

① The total sample size across the 14 included articles comprised 17,122 cases. Within this sample, 1,363 patients were allocated to the experimental group (very low birth weight infants with pulmonary hemorrhage), while the control group (very low birth weight infants without pulmonary hemorrhage) consisted of 15,759 cases.; ② The 14 papers included included 7 papers in English [9, 11–14, 21, 22], 1 paper in French [10], and 6 papers in Chinese [15–20]; ③ The 14 included papers were published in years spanning from 2000 to 2024; all papers reported studies spanning from January 1997 to January 2022; ④ There was one retrospective cohort studies [11] and 13 case-control studies [9, 10, 12–22]; ⑤ The study area of the included literature were from 4 countries (Republic of Korea, France, China, and Germany); ⑥ The 14 included studies identified a total of 36 distinct risk factors. The fundamental characteristics of these studies are summarized in Table 1.

**Table 1:** Basic information of included literature

Serial number	Literature	Study area	Research time	Type of Study	Number of case group	Number of control group	Risk factors
NO. 1	Jong Ki Jung 2024 [9]	Korea University Ansan Hospital, Ansan-si, Republic of Korea	2013–2020	Case control	870	12,956	
NO. 2	M. Erradi 2024 [10]	Service de médecine et réanimation néonatales de Port-Royal, hôpital Cochin, Paris	1st september 2014 to 30th July 2017	Case control	33	33	
NO. 3	Ting-Ting Wang 2019 [11]	Department of Neonatology, Shanghai First Maternity and Infant Hospital, Tongji University School of Medicine	January 1st, 2014 and December 31st, 2017	retrospective cohort studies	30	130	
NO. 4	Jakob Usemann 2017 [12]	Department of Neonatology, Charité–Universitätsmedizin Berlin, Germany	2002年至2012年	Case control	20	40	
NO. 5	Ying-Yao Chen 2012 [13]	Kaohsiung Veterans General Hospital	January 2000 and December 2010	Case control	16	383	
N0. 6	Tsung-Wen Lin 2000 [14]	china medical college hospital, Taichuang, Taiwan	January 1997 to December 1998	Case control	20	20	
NO. 7	Min Qi 2023 [15]	Sir Run Run Shaw Hospital, Zhejiang University School of Medicine	February 2018 to January 2022	Case control	28	86	
NO. 8	Liu Yingying 2023 [16]	NICU, Women and Children’s Hospital, Qingdao University	January 1, 2017 to December 31, 2021	Case control	86	202	
NO. 9	Cao Zhaolan 2022 [17]	Jiangsu Women And Children Health Hospital and Children’s Hospital of Nanjing Medical University	January 1, 2020 to December 31, 2021	Case control	44	530	
NO. 10	Yang Jufen 2018 [18]	Minqin County People’s Hospital, Gansu Province, China	January 2014-June 2017	Case control	24	101	
NO. 11	Chen Dan 2017 [19]	Neonatology Department, Shengjing Hospital of China Medical University	January 2009-December 2015	Case control	35	176	
NO. 12	Cao Mengchen 2017 [20]	Second Neonatology Department, Shengjing Hospital of China Medical University	January 2010-December 2015	Case control	71	364	
NO. 13	Jing Li 2021 [21]	NICU of Hangzhou First People’s Hospital	January 01 2010 and December 31 2019	Case control	51	548	
NO. 14	Jing-jing Pan 2023 [22]	NICU of Children’s Hospital Affiliated to Nanjing Medical University	January 1, 2019 to December 31, 2021	Case control	35	190	

### Literature quality evaluation

All 14 included studies achieved a Newcastle-Ottawa Scale (NOS) score of 7 or higher, indicating high quality. One study (Study No. 10) received a score of 7, while the remaining 13 studies scored 8, further confirming their reliability. The single cohort study (*n* = 1) included in the analysis met nearly all the criteria outlined by the quality assessment tool, with its detailed NOS score provided in Table 2.

**Table 2:** Quality assessment table for cohort studies (NOS)

Serial number	Literature	Type of Research	Selection	Comparabality	Outcome	Totals
NO.3	Ting‑Ting Wang 2019	retrospective cohort studies	☆☆☆	☆☆	☆☆☆	8

**Fig. 2:**
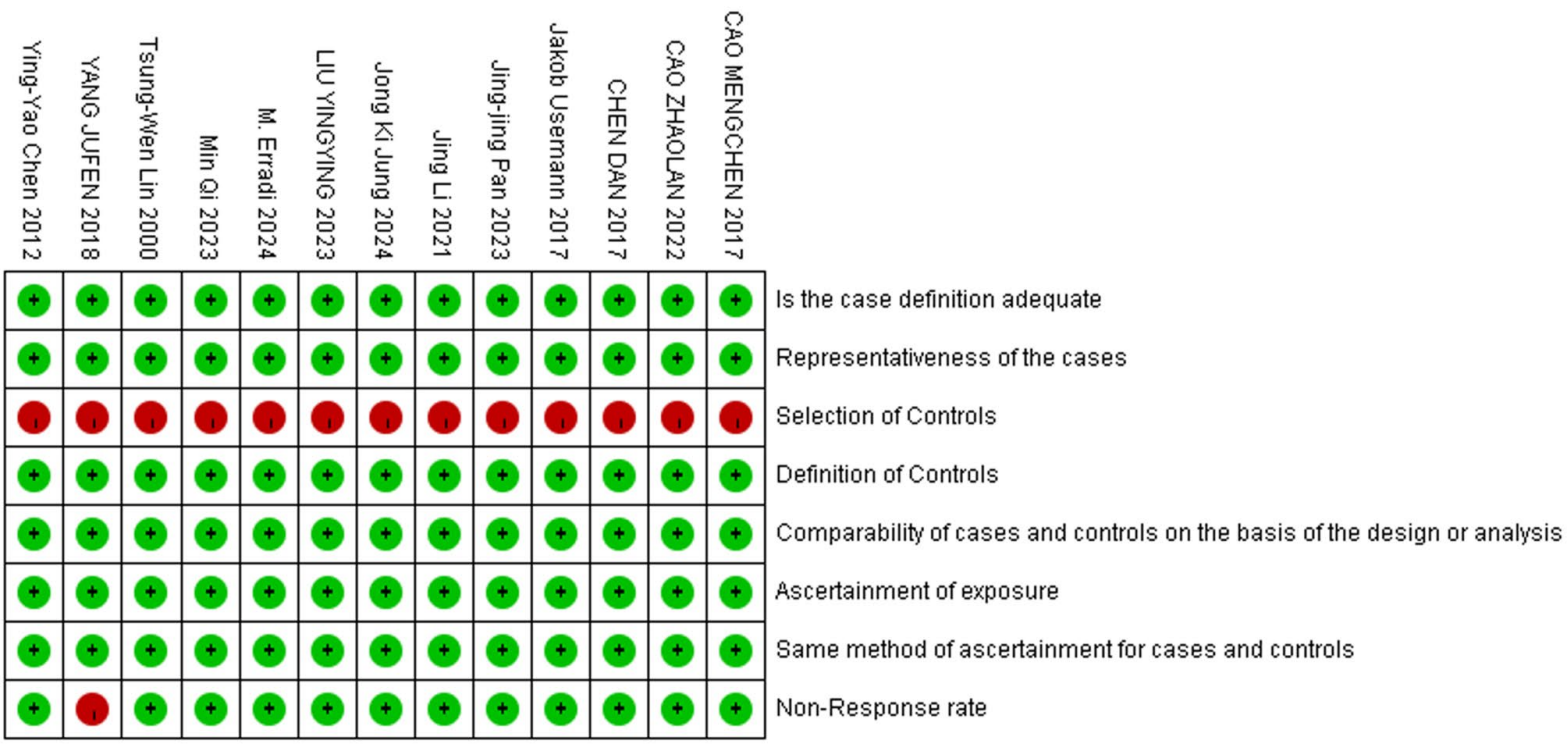
Summary quality assessment chart of the included case-control literature

Among the 13 case-control studies(*n* = 13), most satisfied the majority of quality assessment criteria, though certain items, such as “Selection of Controls” and “Non-Response Rate,” were not fully met. Nonetheless, the overall quality of the included studies was strong. A summarized quality assessment, detailing the distribution of NOS scores, is presented in Fig. 2.

### Meta analysis results

Eleven relevant risk factors for pulmonary hemorrhage in VLBWIs were selected for meta-analysis. The heterogeneity analysis showed that four factors—use of surfactant, respiratory distress syndrome (RDS), endotracheal intubation in the delivery room, and male—demonstrated low heterogeneity across the studies. These factors were analyzed using a fixed-effects model. In contrast, other factors, such as patent ductus arteriosus (PDA), low 5-minute Apgar score, complete course of antenatal corticosteroids(ACS), birthweight, gestational age, small for gestational age (SGA), and early-onset sepsis (EOS), exhibited greater heterogeneity and were analyzed using a random-effects model.

**Fig. 3:**
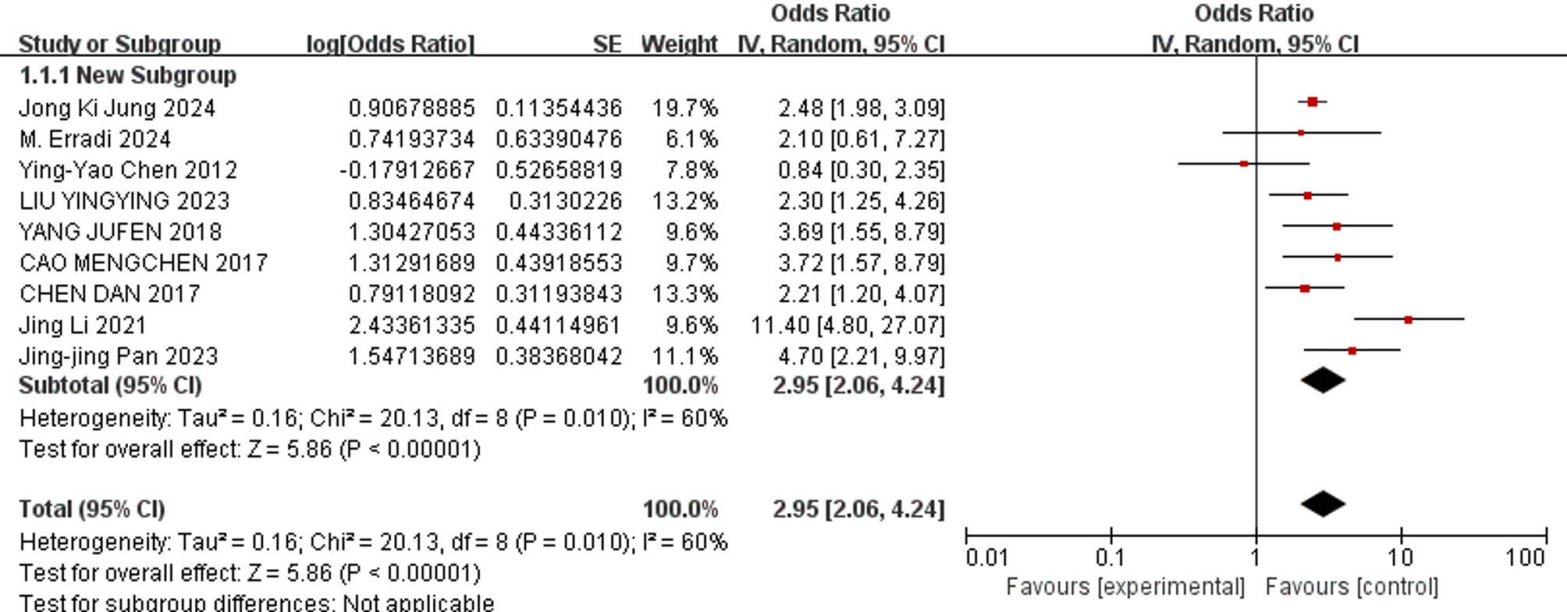
Forest plot of the relationship between PDA and the incidence of pulmonary hemorrhage of very low birth weight infants

**Fig. 4:**
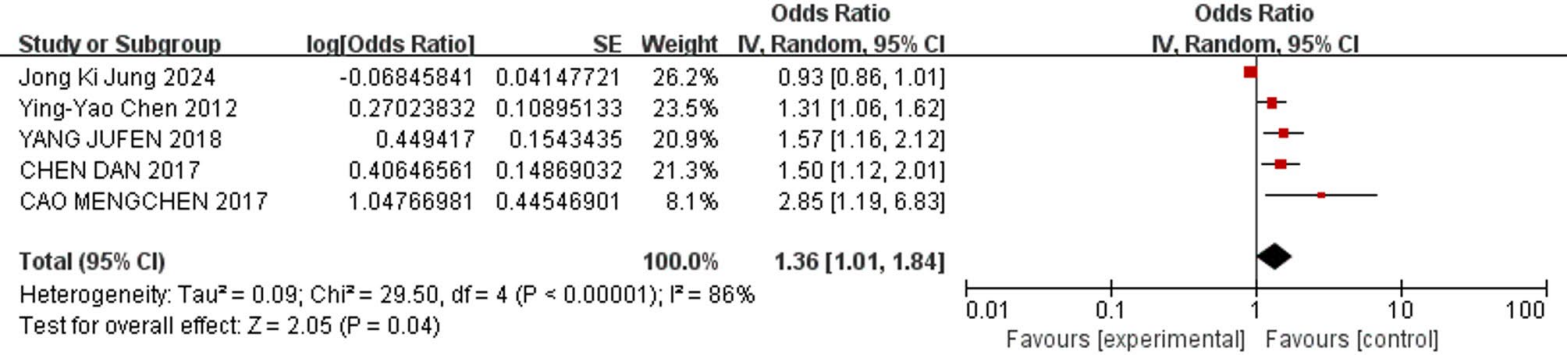
Forest plot of the relationship between low 5-min Apgar score and the incidence of pulmonary hemorrhage of very low birth weight infants

**Fig. 5:**
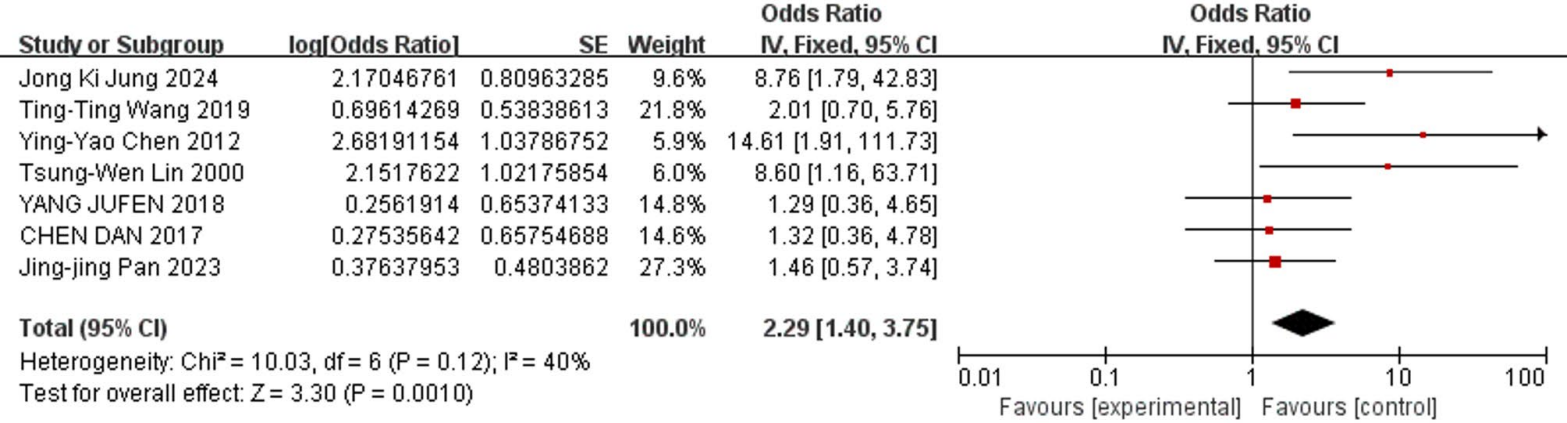
Forest plot of the relationship between Use of surfactant and the incidence of pulmonary hemorrhage of very low birth weight infants

**Fig. 6:**
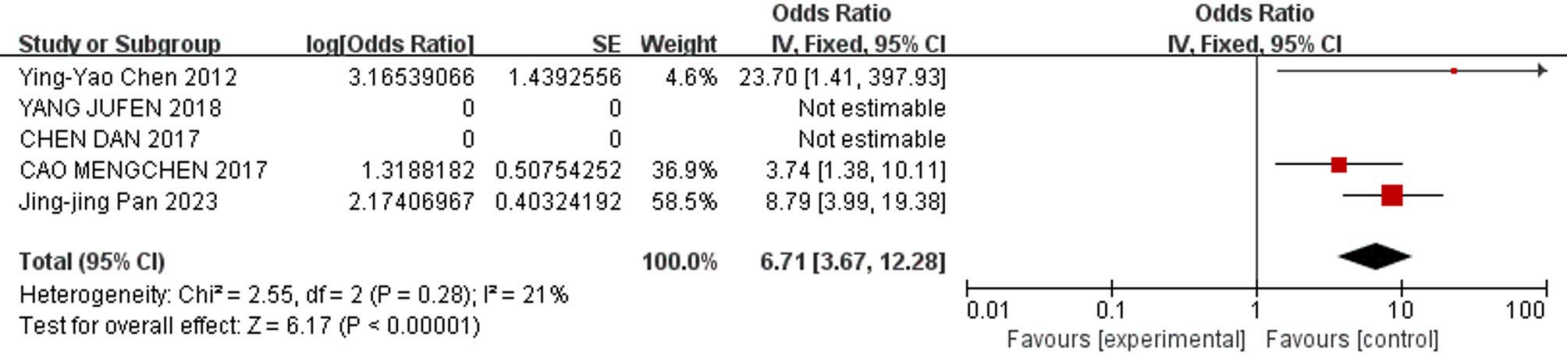
Forest plot of the relationship between RDS and the incidence of pulmonary hemorrhage of very low birth weight infants

**Fig. 7:**
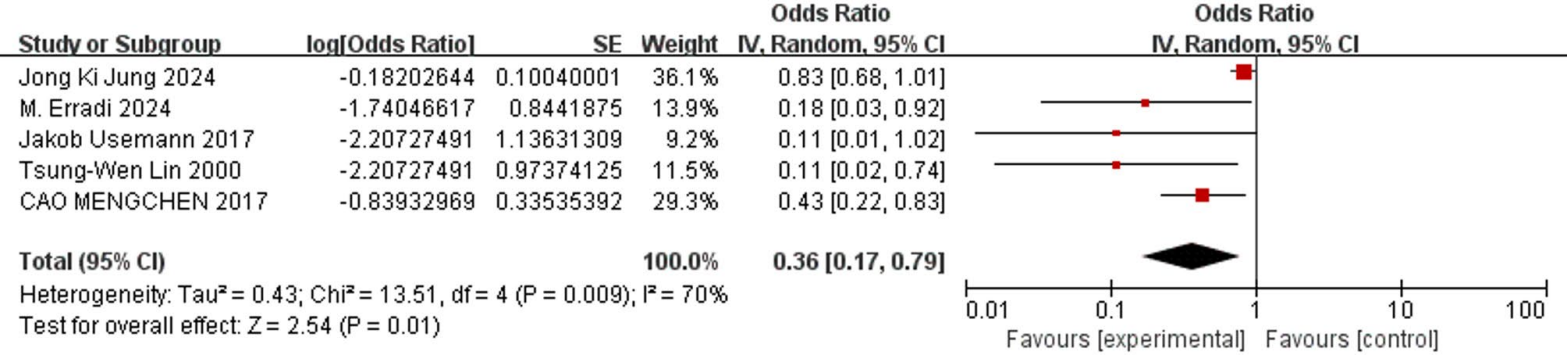
Forest plot of the relationship between complete course of antenatal corticosteroids and the incidence of pulmonary hemorrhage of very low birth weight infants

**Fig. 8:**
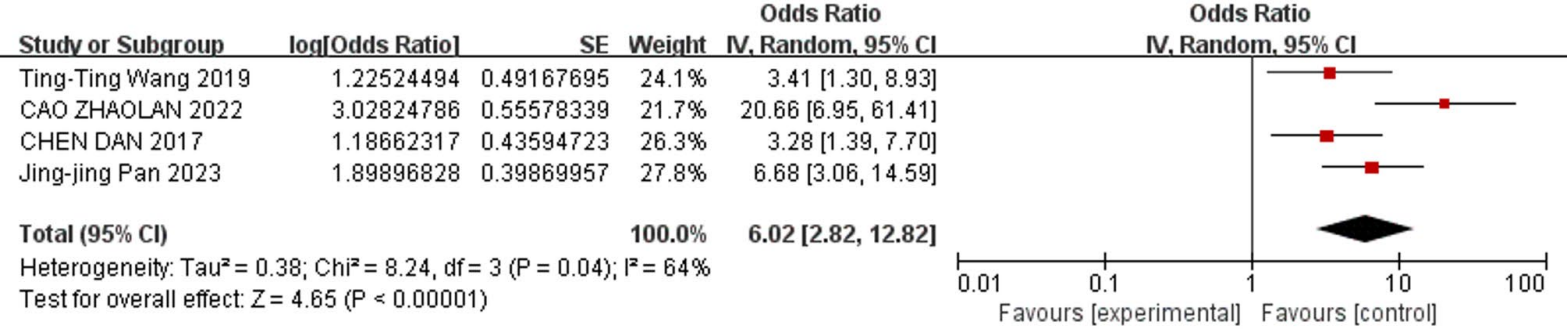
Forest plot of the relationship between early-onset sepsis and the incidence of pulmonary hemorrhage of very low birth weight infants

The meta-analysis revealed the following results: The combined odds ratios for endotracheal intubation in the delivery room, male sex, birthweight, gestational age, and SGA were not statistically significant (*P* ≥ 0.05); Conversely, the combined odds ratios for patent ductus arteriosus, low 5-minute Apgar score, use of surfactant, respiratory distress syndrome, complete course of ACS, and early-onset sepsis (EOS) were statistically significant (*P* < 0.05). The detailed results of the heterogeneity test and meta-analysis are provided in Table 3 and illustrated in Figs. 3, 4, 5, 6, 7 and 8.

**Table 3:** Results of heterogeneity test and meta analysis of pulmonary hemorrhage risk factors

NO	Research factors	Literature source	Heterogeneity test			Effect model	merge OR (95%CI)	merge *P* value
			Q	I ^2^	*P*			
NO. 1	Patent ductus arteriosus	[9, 10, 13, 16, 17–22]	6.13	2	0.41	Random effect	2.95 [2.06–4.24]	*P* < 0.00001
NO. 2	low 5-min Apgar score	[9, 13, 18–20]	29.5	86	*P* < 0.00001	Random effect	1.36 [1.01–1.84]	0.04
NO. 3	Use of surfactant	[9, 11, 13, 14, 18, 19, 22]	10.03	40	0.12	Fixed effect	2.29 [1.40–3.75]	0.001
NO. 4	Respiratory Distress Syndrome	[13, 18–20, 22]	2.55	21	0.28	Fixed effect	6.71 [3.67–12.28]	*P* < 0.00001
NO. 5	Complete antenatal corticosteroid	[9, 10, 12, 14, 20]	13.51	70	0.009	Random effect	0.36 [0.17–0.79]	0.01
NO. 6	Early-onset sepsis	[11, 17, 19, 22]	8.24	64	0.04	Random effect	6.02 [2.82–12.82]	*P* < 0.00001
NO. 7	(Endotracheal intubation in the delivery room	[10, 11, 20]	0.44	0	0.8	Fixed effect	1.05 [0.62–1.77]	0.86
NO. 8	birthweight	[13, 18–20]	26.97	89	*P* < 0.00001	Random effect	0.99 [0.98- 1.00]	0.07
NO. 9	Male	[9, 12, 20]	2.41	17	0.3	Fixed effect	1.10 [0.92–1.31]	0.3
NO. 10	gestational age	[9, 11, 13, 18–20]	15.86	68	0.007	Random effect	0.94 [0.88–1.01]	0.09
NO. 11	small for gestational age	[9, 11, 20]	4.11	51	0.13	Random effect	1.34 [0.68–2.64]	0.4

### Sensitivity analysis

The sensitivity analysis, conducted by comparing the results of the fixed-effects model and the random-effects model. Except for the risk factor of low 5-minute Apgar score, the combined effect values of the other risk factors of the two models did not show significant differences. And the calculated results were relatively stable. We used the one-by-one elimination method to explore the risk factor of low 5-minute Apgar score. It was found that the heterogeneity decreased significantly after excluding Reference [9] (accounting for 26.2% of the weight). After removing this reference, a re-analysis of the remaining risk factors using the fixed effects model showed consistent results and no changes (OR 1.45, 95% CI: 1.25–1.68, *P* < 0.00001), indicating that the pooled results of this study are fundamentally stable. Detailed information is shown in Table 4.

**Table 4:** Sensitivity analysis and publication bias test

Research factors	Sensitivity analysis			Egger’s test			Begg’s test
	Fixed effects model OR(95%CI)	Random effects model OR(95%CI)		T value		*P* value	*P* value
Patent ductus arteriosus	2.68 [2.25, 3.19]		2.95 [2.06–4.24]]	0.65	0.534		0.602
low 5-min Apgar scorea	1.03 [0.96–1.11]		1.36 [1.01–1.84]	6.77	0.007		0.462
Use of surfactantb	2.29 [1.40–3.75]		2.61 [1.34–5.06]	3.43	0.019		0.035
Respiratory Distress Syndrome	6.71 [3.67–12.28]		6.69 [3.20-13.98]	0.38	0.769		1.000
Complete antenatal corticosteroidc	0.75 [0.62–0.90]		0.36 [0.17–0.79]	−13.05	0.001		0.462
Early-onset sepsis	5.74 [3.66-9.00]		6.02 [2.82–12.82]	0.78	0.516		0.734

Note: a means that the corrected OR and 95% CI are 1.364(1.014–1.836);B means that the corrected OR and 95% CI are 1.364༈1.014–1.836) 2.250 ༈1.108–4.569). C means that the corrected OR and 95% CI are 1.364༈1.014–1.836) 0.364༈0.167–0.794)

### Publication bias

**Fig. 9:**
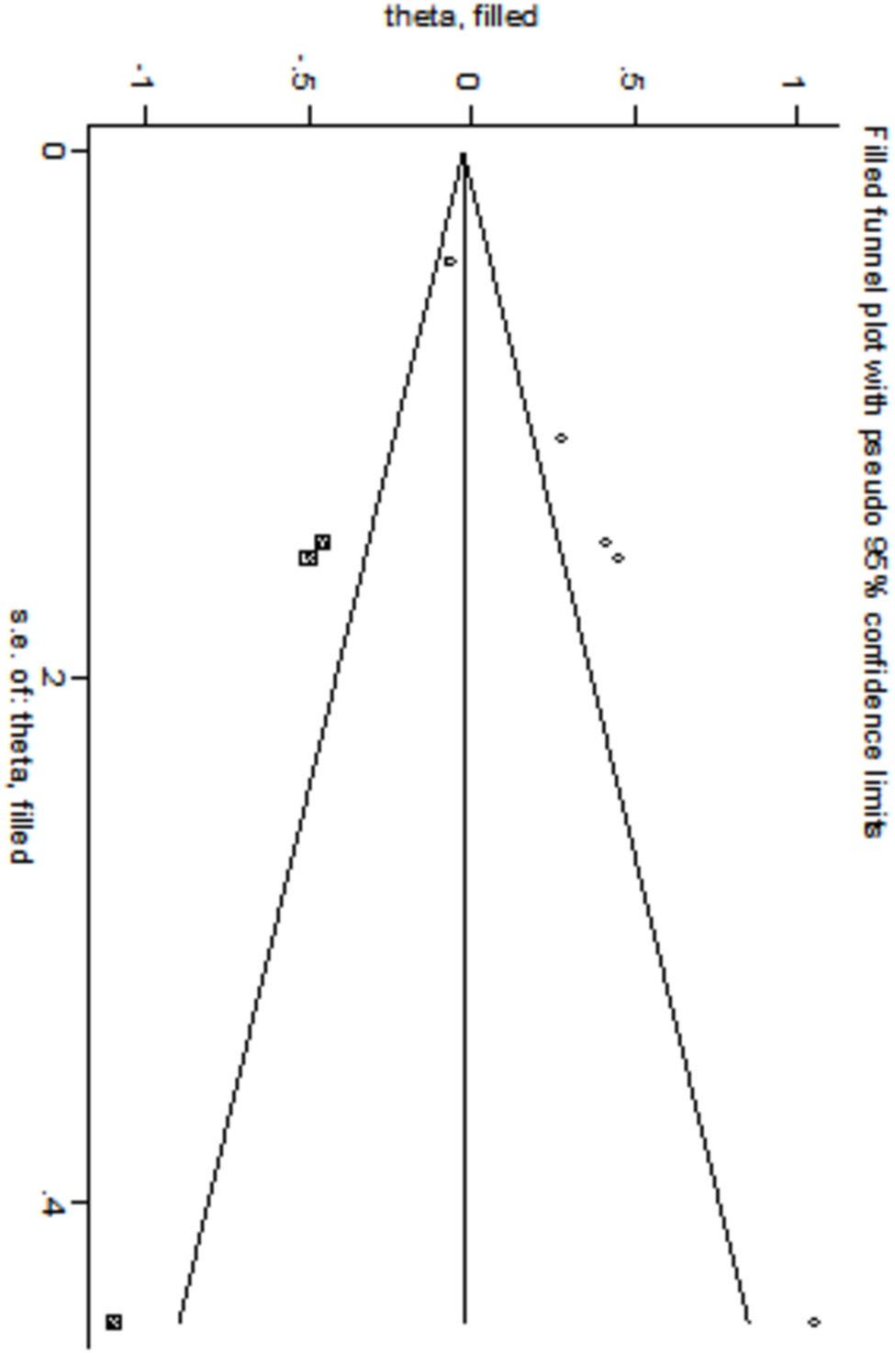
Low 5-min Apgar score cut-and-patch diagram

**Fig. 10:**
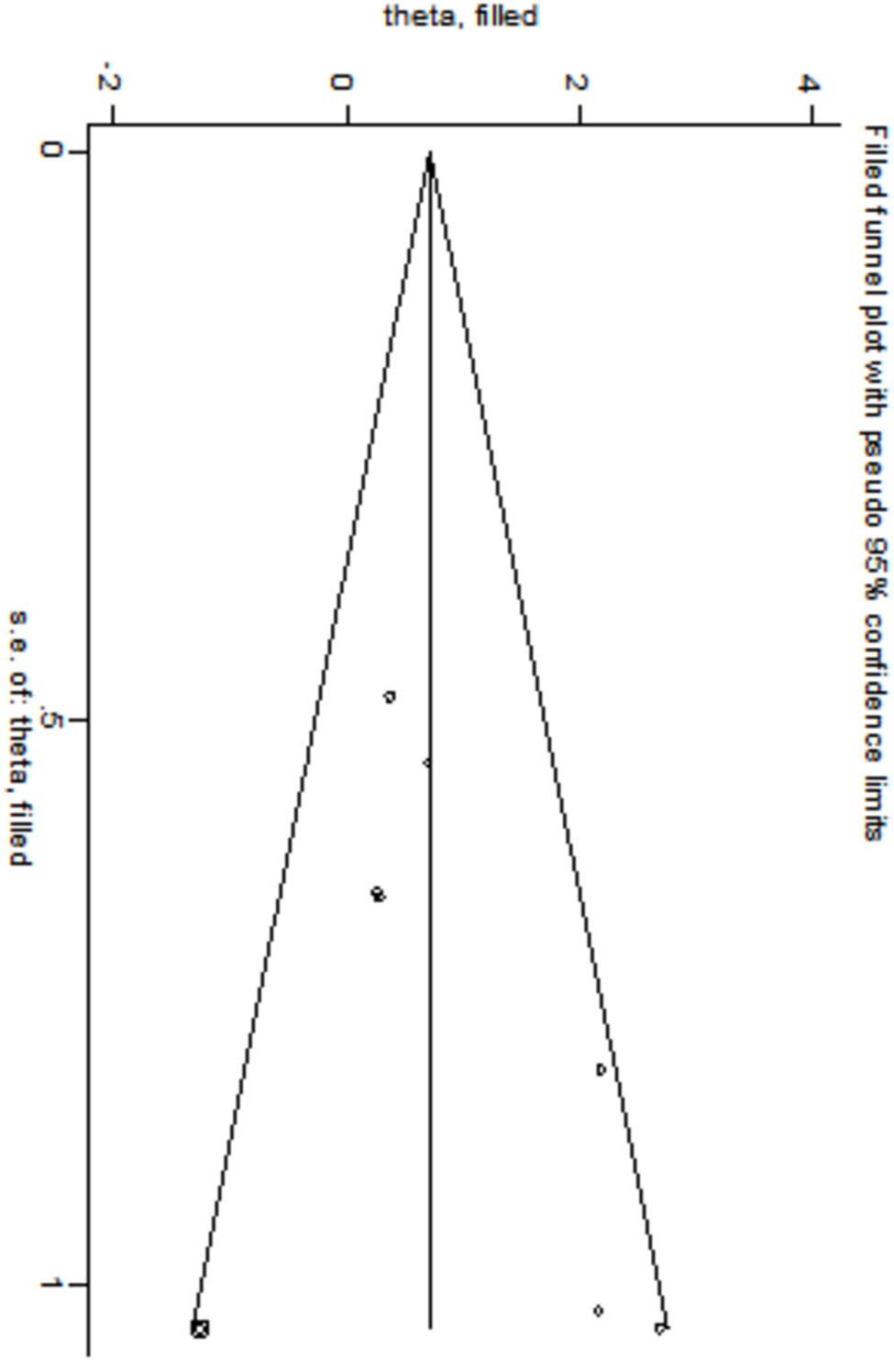
Use of surfactant cut-and-patch diagram

Egger’s and Begg’s bias tests were employed to evaluate the risk of bias for each factor. The results of Egger’s test indicated significant publication bias for the following factors: low 5-minute Apgar score (*t* = -6.77, *P* = 0.007), use of surfactant (*t* = 3.43, *P* = 0.019), and complete course of ACS (*t* = -13.05, *P* = 0.001). These findings are presented in Table 4, which has been adjusted using the cut-and-patch method to account for the identified biases in the three factors (low 5-minute Apgar score, use of surfactant, and complete course of ACS).The results indicated that: ① After adjusting for low 5-minute Apgar scores, the odds ratio slightly increased to 1.364 (95% CI: 1.014–1.836), although the direction remained unchanged, as illustrated in the shear-and-patch plot in Fig. 9. ② After adjusting for the use of surfactant, the OR also slightly increased to 2.250 (95% CI: 1.108–4.569), with the direction remaining consistent, as depicted in Fig. 10. ③ After adjusting for complete course of ACS, the OR slightly decreased to 0.364 (95% CI: 0.167–0.794), but the direction remained unchanged.

**Fig. 11:**
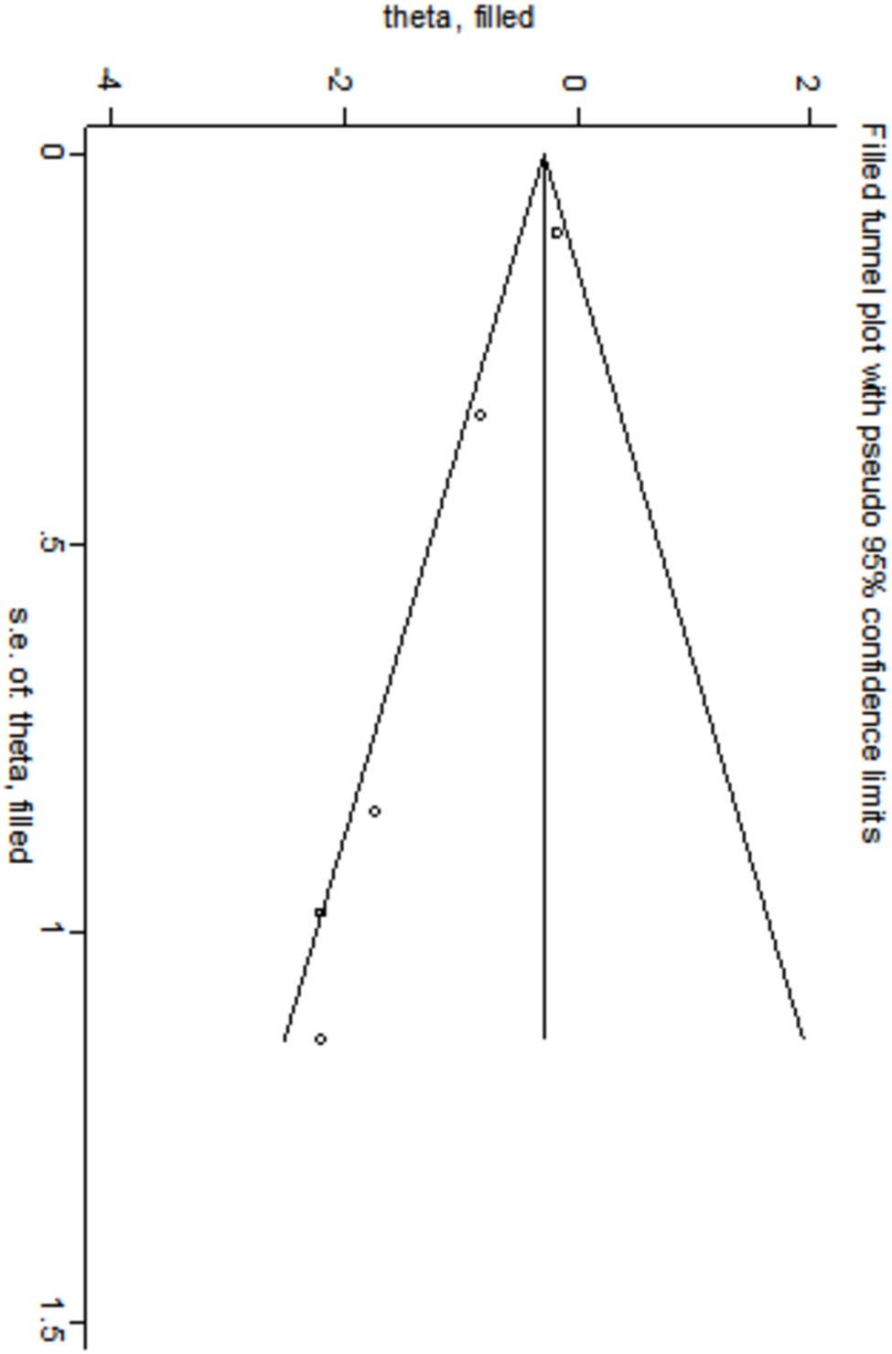
Complete course of ACS cut-and-patch diagram

The cut-and-patch plot presented in Fig. 11 demonstrates that the outcomes of the three risk factor analyses were both stable and reliable. For the remaining risk factors, the results indicated *P* > 0.05, suggesting the absence of publication bias. The Egger’s test and Begg’s test results of all risk factors are detailed in Table 4.

## Discussion

Pulmonary hemorrhage in very low birth weight infants is influenced by various perinatal factors, making early diagnosis challenging but critical for prevention. Clarifying the high risk factors of pulmonary hemorrhage serves the prevention of the disease. This study explored the risk factors for pulmonary hemorrhage and conducted a meta-analysis of 36 risk factors from 14 studies. Meta-analysis showed that risk factors such as patent ductus arteriosus, low 5-minute Apgar scores, use of surfactant, respiratory distress syndrome, early-onset sepsis, are associated with an increased risk of pulmonary hemorrhage in VLBW infants. In contrast, complete course of antenatal corticosteroids has a protective effect in reducing the risk of this severe complication.

The results of this study showed that PDA was a risk factor for pulmonary hemorrhage of VLBWIs (OR = 2.95). Pulmonary hemorrhage is strongly correlated with the altered hemodynamic status associated with left-to-right shunting through the ductus arteriosus [27]. This relationship was first reported in 1994 in a retrospective cohort study published by Garland and associates [28]. Use of surfactant also was a risk factor (OR = 2.29). The introduction of surfactant therapy has significantly improved neonatal outcomes in RDS management, yet paradoxically, it has been linked to an increased risk of PH in certain high-risk populations. As reported in a recent meta-analysis [29], surfactant treatment is associated with an increased risk of PH in preterm infants. Those may be related to the following mechanisms. First of all, in immediate postnatal life, very low birth weight infants with an immature circulatory system experience a transition from fetal to postnatal circulation, with dilation of the small pulmonary arteries, a rapid decrease in pulmonary vascular resistance, a left-to-right shunt at the level of the ductus arteriosus. This shunt exposes the immature pulmonary vasculature to systemic pressure. The increase in blood flow to the pulmonary beds engorges the thin pulmonary capillaries [30]. Secondly, for very low birth weight infants with PDA, pulmonary vascular resistance decreases rapidly after surfactant treatment, resulting in a sudden increase in arterial duct blood flow (an increased pulmonary-to-systemic flow ratio [Qp/Qs]). This sudden hemodynamic and compliance change increases the risk of capillary rupture and the loss of epithelial and endothelial integrity and bleeding [29]. These processes can give rise to pulmonary edema, microvascular injury, and terminal capillary hemorrhage [31]. Thirdly, the walls of pulmonary capillaries are inherently very thin. Pulmonary capillary pressure under ventilation increases further when pulmonary surfactant (PS) is applied to increase lung compliance. The stress of the pulmonary capillary wall becomes very large, and the ultra-structural damage of the pulmonary capillary wall occurs. This process can lead to hyperpermeability pulmonary edema and even direct hemorrhage [32, 33]. Fourthly, an in vitro study has shown that the presence of pulmonary surfactant can impair coagulation function [34], but this finding has not yet been confirmed in the clinical setting.

We consulted the latest guidelines and reviewed the literature we included. Current treatment approaches for patent ductus arteriosus in VLBWIs remain controversial, with the relative merits of active treatment versus expectant management yet to be conclusively established [35, 36]. Included literature of our study state that haemodynamically significant PDA needs to be managed by neonatologist, with ibuprofen administered to close the ductus arteriosus and surgical closure performed when necessary. All Included literature emphasised the need to minimise unnecessary surfactant use, avoid prophylactic administration, and employ selective treatment only for infants with clinically confirmed RDS. However, specific protocols for distinguishing between prophylactic and rescue administration (such as dosage and formulation) were not delineated. Based on the above mchanisms, after consulting relevant materials and literature, our team has summarised the following: First, PDA requires a comprehensive management strategy, one aspect of which is appropriate respiratory support. Appropriate PEEP(6 ~ 8 cmH _2_O) should be given after the birth of VLBWIs to reduce pulmonary stasis and pulmonary edema, and thus reduce the chance of pulmonary hemorrhage [36–38].Afterwards, an appropriate level of PEEP can help prevent PH by modulating the capacitance of the pulmonary vascular bed. By increasing the end-expiratory lung volume (EELV), it may enhance parenchymal expansion and thereby improve vascular compliance. Conversely, positive pressure can also exert a protective effect by partially compressing pulmonary vessels and limiting hyperperfusion, as may occur in the presence of a patent ductus arteriosus or following surfactant administration. Second, surfactant should be administered in the early phases of RDS to VLBWIs who exhibit clinical signs of the disease, while avoiding its prophylactic use through intubation, in accordance with international guidelines [38, 39]. Nevertheless, ongoing studies are expected to further clarify variables such as the optimal timing and therapeutic dose of surfactant administration [40].

The results of this study showed that RDS was a risk factor for pulmonary hemorrhage of very low birth weight infants (OR = 6.71). Recent studies indicate that pulmonary hemorrhage is more prevalent among patients with respiratory distress [29]. Infants who experienced respiratory distress shortly after birth, are more likely to develop pneumonedema. In these situations, the tension imposed secondary to fluid and erythrocyte accumulation within the alveolus increases alveolar epithelial permeability [2, 41]. The increase in permeability permits the infiltration of cellular defenses linked to the innate immune response. These cells include neutrophils and proinflammatory cytokines. Over time, junctions between alveolar cells are degraded and the epithelial wall ruptures, allowing the migration of fluid and erythrocytes upward and into the anatomic air spaces. Therefore, VLBWIs with RDS should be treated aggressively to minimize the impact on pulmonary hemorrhage [38].

The results of this study showed that sepsis was a risk factor for pulmonary hemorrhage in VLBWIs (OR = 1.36). PH may result from profound microvascular injury and inflammation [8]. In VLBWIs with immature pulmonary vasculature, the synergy between sepsis-induced microvascular permeability, endothelial damage, coagulopathy, and fragile capillaries creates a critical environment that is highly conducive to PH. Any severe sepsis (also caused by Gram-positive bacteria) can lead to coagulopathy and increase the risk of bleeding, especially in preterm or critically ill neonates. Even if Gram-negative sepsis, due to its ability to trigger a more intense inflammatory and coagulopathic response (Disseminated Intravascular Coagulation, DIC), can further increase bleeding risk [42]. For neonatal sepsis, early identification and assessment, prompt treatment, and active investigation of the source of infection are essential [43].

The study revealed that low 5-minute Apgar scores was a risk factor of pulmonary hemorrhage in VLBWIs (OR = 1.36), suggesting that low 5-minute Apgar scores may indicate a higher risk of PH. The apgar scores is an important indicator of asphyxia. Asphyxia develops secondary to a significant, prolonged deficit of oxygen delivery to the tissues. Lung injury occurs in 25% of all asphyxiated newborns, which ranges from a mild respiratory distress to pulmonary hemorrhage/severe respiratory failure [44]. The persistent state of insufficient oxygenation leads to left ventricular failure, and as cardiac afterload rises, an increase in pulmonary capillary pressure develops. The increase in pulmonary capillary pressure leads to fluid congestion within the lungs, which serves as a driving force for fluid and erythrocyte migration toward the lung interstitium [45]. A study in the UK in 2014 showed that the incidence rate of low 5-minute Apgar score was 15.4 per thousand [46]. Whereas a study in China in 2022 reported the incidence rate of low 5-minute Apgar scores was 3.9 per thousand [47]. It is recommended that all VLBW infants receive resuscitation measures in the delivery room that initiate respiratory support within the first minute of life, to guarantee proper oxygenation of the myocardium, and ensure its adequacy throughout the entire resuscitation period.

The results of this study showed that complete course of antenatal corticosteroids was a protective factor of pulmonary hemorrhage in VLBWIs (OR = 0.36), suggesting that complete course of antenatal corticosteroids can reduce the incidence of neonatal pulmonary hemorrhage. This protective effect is likely due to the role of antenatal steroids in promoting lung maturation, enhancing surfactant production, and stabilizing the pulmonary vasculature [48]. A large data collected prospectively for the Neonatal Research Network Generic Database [49] showed that the incidence of pulmonary hemorrhage did not differ by any ACS exposure but the incidence of death due to pulmonary hemorrhage was significantly decreased by a complete course of ACS compared to a partial course of antenatal corticosteroids. Antenatal steroids might not have been given due to the necessity of rapid delivery as a result of maternal illness or fetal distress. Although not reflected by higher CRIB scores, the reduced incidence of PH in response to steroids may result, on the cellular level, from enhanced microvascular maturation and premature focal capillary fusion [50]. Berger et al. [48] reported in 2000 that prenatal glucocorticoid use was a protective factor against the development of pulmonary hemorrhage, especially in neonates with gestational age between 24 and 26 weeks. In addition, Kluckow et al. [51] observed lower glucocorticoid coverage in neonates with pulmonary hemorrhage. Guidelines recommend routine administration of one course of ACS at 34 weeks of gestation for singleton pregnancies at risk of preterm delivery. For women at risk of late preterm delivery at 34–36 weeks of gestation, ACS should be used with caution after a comprehensive assessment of the pros and cons of various options [52]. At present, the gestational period for the use of this drug has been clearly defined. The included literatures all defaulted to the gestational age range and administration regimens of ACS as defined in the guidelines. Efforts should be made to increase the coverage rate of antenatal steroid administration for all women at risk of preterm birth, in every healthcare setting.

The advantages of our study are as follows. Firstly, this is the first meta-analysis to specifically address risk factors for pulmonary hemorrhage of very low birth weight infants. This further enhances understanding of the risk factors associated with this condition. Secondly, more than 1300 articles were screened for eligibility and 14 studies were included with more than 17,000 total participants in the meta-analysis, which greatly increased the robustness of our results.Thirdly, the study used extensive methods to synthesise as many individual study results as possible. Particularly, it harmonised outcome measures and exposure units to facilitate statistical analysis.Finally, comprehensive sensitivity, and publication bias analyses were conducted to elucidate the detected interstudy heterogeneity. Furthermore, to mitigate the occurrence of pulmonary haemorrhage, innovative insights and measures have been summarised for each risk factor in the discussion section after consulting relevant materials and literature. In-depth analyses have been conducted on risk factors such as respiratory distress syndrome (RDS), patent pulmonary ductus (PDA), and the use of surfactants, as well as the combined application of these factors. The factors identified in the conclusions are not non-modifiable factors (such as gestational age, genetic background, fetal growth restriction), but rather modifiable factors (such as PDA management, timing of surfactant, antenatal corticosteroid use, and sepsis management), to concentrate resources on interventions that can be realistically altered at the bedside.

However, there are some limitations in this study. Firstly, inter-study heterogeneity may affect the interpretation of our findings. The high heterogeneity of some of risk factors in this study may be explained by the following: On the one hand, the types of literature included were case-control studies and cohort studies, and there were methodological differences between the studies.On the other hand, clinical heterogeneity exists because of the inconsistency in the timing of the studies across the literature and the possibility that differences in racial or ethnic background may be contributed to the variability observed across regions. However, our sensitivity analyses (comparing the results of the fixed-effects model and the random-effects model, one-by-one elimination method) indicate that the pooled results of this study remain fundamentally robust. Secondly, the type of design of the included literature was mostly case-control studies, and selection bias and recall bias were difficult to avoid. To enhance comprehensibility and validity, we predefined the individual quality criteria. The literature search was primarily conducted by two investigators with a comprehensive hand-search of reference lists. The quantitative analysis included studies that were published from database inception until May 20, 2024. Thirdly, studies were all published in English, French, and Chinese, which may have potential publication bias and language bias; therefore, future studies should include a wider range of languages. Fourthly, this study included literature research sites from four countries (regions), with limited data sources. Future studies should include more regions.

## Conclusion

In conclusion, this meta-analysis evaluated the risk factors for pulmonary hemorrhage of very low birth weight infants and their correlation values, in which patent ductus arteriosus, low 5-minute Apgar scores, use of surfactant, respiratory distress syndrome, early-onset sepsis may increase the risk of pulmonary hemorrhage development, while complete course of antenatal corticosteroid administration reduces the risk of pulmonary hemorrhage of very low birth weight infants. In addition, no correlation was discovered between PH in VLBWIs and resuscitation by tracheal intubation in the delivery room, birth weight, male, gestational age, and small for gestational age. Therefore, early recognition of PDA, prevention of neonatal asphyxia, rational use of alveolar surface-active substances, and appropriate treatment of respiratory diseases and infections should be used to prevent the occurrence of pulmonary hemorrhage of very low birth weight infants. Future research should explore early risk stratification models based on cardiac ultrasound (such as strain-velocity relationships), heart rate variability, near-infrared spectroscopy, and other modalities for precise prediction. Efforts should be made to promote the standardised application of pulmonary ultrasound and cardiac ultrasound in the diagnosis and monitoring of PH.

## Supplementary Information

Below is the link to the electronic supplementary material.

Supplementary Material 1: Search strategy

Supplementary Material 2: Data extracted from included studies

## Data Availability

All data are fully available without restriction.All relevant data are within the manuscript and its Supporting Information files.

## Abbreviations

ACS: Antenatal corticosteroids
BPD: Bronchopulmonary dysplasia
BW: Birth weight
CI: Confidence intervals
DIC: Disseminated intravascular coagulation
GA: Gestational age
NICU: Neonatal intensive care unit
OR: Odds ratio
PDA: Patent ductus arteriosus
PH: Pulmonary hemorrhage
PRISMA: Preferred Reporting Items for Systematic Reviews and Meta-Analyses
PS: Pulmonary surfactant
RDS: Respiratory Distress Syndrome
SGA: Small for gestational age
VLBW: Very low birth weight
VLBWIs: Very low birth weight infants
