# Supplementary material for: Risk factors for pulmonary hemorrhage of very low birth weight infants: a meta-analysis
Source: Ital J Pediatr. 2026 Feb 3;52:33. doi: 10.1186/s13052-025-02194-2 (PMC12958614; doi:10.1186/s13052-025-02194-2)
Supplement: Supplementary file 1 — Supplementary Material 1: Search strategy [file 13052_2025_2194_MOESM1_ESM.doc]

1、Patent ductus arteriosus

| NO | author | OR | OR_LL | OR_UL | Ln or | SE |
| --- | --- | --- | --- | --- | --- | --- |
| 1 | Jong Ki Jung 2024 | 2.476357803 | 1.990056633 | 3.105755459 | 0.906788853 | 0.11354436 |
| 2 | M. Erradi 2024 | 2.1 | 0.6 | 7.2 | 0.741937345 | 0.633904758 |
| 5 | Ying-Yao Chen 2012 | 0.836 | 0.298 | 2.348 | -0.179126666 | 0.526588187 |
| 8 | LIU YINGYING 2023 | 2.304 | 1.238 | 4.223 | 0.834646743 | 0.313022602 |
| 10 | YANG JUFEN 2018 | 3.685 | 1.601 | 9.103 | 1.304270526 | 0.443361121 |
| 11 | CHEN DAN 2017 | 3.717 | 1.572 | 8.793 | 1.312916891 | 0.439185525 |
| 12 | CAO MENGCHEN 2017 | 2.206 | 1.205 | 4.093 | 0.791180921 | 0.311938426 |
| 13 | Jing Li 2021 | 11.4 | 4.79 | 27 | 2.433613355 | 0.441149606 |
| 14 | Jing-jing Pan 2023 | 4.698 | 2.215 | 9.967 | 1.547136886 | 0.383680416 |

2、low 5-min Apgar score

| NO | author | OR | OR_LL | OR_UL | Ln or | SE |
| --- | --- | --- | --- | --- | --- | --- |
| 1 | Jong Ki Jung 2024 | 0.933832292 | 0.860881953 | 1.012874931 | -0.068458415 | 0.041477205 |
| 5 | Ying-Yao Chen 2012 | 1.31027668 | 1.058331612 | 1.622199467 | 0.270238321 | 0.108951328 |
| 10 | YANG JUFEN 2018 | 1.567398119 | 1.109877913 | 2.032520325 | 0.449416996 | 0.154343505 |
| 11 | CHEN DAN 2017 | 1.501501502 | 1.121076233 | 2.008032129 | 0.406465608 | 0.14869032 |
| 12 | CAO MENGCHEN 2017 | 2.851 | 1.191 | 6.828 | 1.04766981 | 0.445469009 |

3、Use of surfactant

| NO | author | OR | OR_LL | OR_UL | Ln or | SE |
| --- | --- | --- | --- | --- | --- | --- |
| 1 | Jong Ki Jung 2024 | 8.762380425 | 3.016841892 | 72.09403682 | 2.170467606 | 0.809632852 |
| 3 | Ting‑Ting Wang 2019 | 2.006 | 0.698 | 5.76 | 0.69614269 | 0.538386135 |
| 5 | Ying-Yao Chen 2012 | 14.613 | 1.911 | 111.728 | 2.681911543 | 1.037867521 |
| 6 | Tsung-Wen Lin 2000 | 8.6 | 1.16 | 63.67 | 2.151762203 | 1.021758543 |
| 10 | YANG JUFEN 2018 | 1.292 | 0.371 | 4.812 | 0.256191405 | 0.65374133 |
| 11 | CHEN DAN 2017 | 1.317 | 0.363 | 4.779 | 0.275356423 | 0.657546879 |
| 14 | Jing-jing Pan 2023 | 1.457 | 0.568 | 3.734 | 0.376379527 | 0.4803862 |

4、Respiratory Distress Syndrome

| NO | author | OR | OR_LL | OR_UL | Ln or | SE |
| --- | --- | --- | --- | --- | --- | --- |
| 5 | Ying-Yao Chen 2012 | 23.698 | 1.411 | 397.892 | 3.165390656 | 1.439255597 |
| 10 | YANG JUFEN 2018 | 0 | 0 | 0 | #NUM! | #NUM! |
| 11 | CHEN DAN 2017 | 0 | 0 | 0 | #NUM! | #NUM! |
| 12 | CAO MENGCHEN 2017 | 3.739 | 1.383 | 10.113 | 1.318818196 | 0.507542518 |
| 14 | Jing-jing Pan 2023 | 8.794 | 3.99 | 19.385 | 2.174069671 | 0.403241924 |

5、Endotracheal intubation in the delivery room

| NO | author | OR | OR_LL | OR_UL | Ln or | SE |
| --- | --- | --- | --- | --- | --- | --- |
| 2 | M. Erradi 2024 | 0.8 | 0.2 | 2.9 | -0.223143551 | 0.682180778 |
| 3 | Ting‑Ting Wang 2019 | 1.508 | 0.389 | 5.844 | 0.41078427 | 0.691222303 |
| 12 | CAO MENGCHEN 2017 | 1.027 | 0.549 | 1.921 | 0.026641931 | 0.319516 |

6、Complete antenatal corticosteroid

| NO | author | OR | OR_LL | OR_UL | Ln or | SE |
| --- | --- | --- | --- | --- | --- | --- |
| 1 | Jong Ki Jung 2024 | 0.833579299 | 0.686230098 | 1.017171523 | -0.182026442 | 0.100400012 |
| 2 | M. Erradi 2024 | 0.175438596 | 0.033222591 | 0.909090909 | -1.740466175 | 0.844187498 |
| 4 | Jakob Usemann 2017 | 0.11 | 0.01 | 0.86 | -2.207274913 | 1.136313086 |
| 6 | Tsung-Wen Lin 2000 | 0.11 | 0.017 | 0.773 | -2.207274913 | 0.973741251 |
| 12 | CAO MENGCHEN 2017 | 0.432 | 0.224 | 0.834 | -0.839329691 | 0.335353916 |

7、birthweight

| NO | author | OR | OR_LL | OR_UL | Ln or | SE |
| --- | --- | --- | --- | --- | --- | --- |
| 5 | Ying-Yao Chen 2012 | 0.739591083 | 0.62254289 | 0.878646241 | -0.301657836 | 0.087900468 |
| 10 | YANG JUFEN 2018 | 0.969 | 0.993 | 1.023 | -0.031490667 | 0.007592883 |
| 11 | CHEN DAN 2017 | 0.999 | 0.995 | 1.002 | -0.0010005 | 0.001788404 |
| 12 | CAO MENGCHEN 2017 | 0.998 | 0.996 | 1 | -0.002002003 | 0.001022454 |

8、Male

| NO | author | OR | OR_LL | OR_UL | Ln or | SE |
| --- | --- | --- | --- | --- | --- | --- |
| 1 | Jong Ki Jung 2024 | 1.077 | 0.893 | 1.299 | 0.074179398 | 0.095602917 |
| 4 | Jakob Usemann 2017 | 0.44 | 0.09 | 2.21 | -0.820980552 | 0.816565848 |
| 12 | CAO MENGCHEN 2017 | 1.507 | 0.838 | 2.71 | 0.41012092 | 0.299409646 |

9、Gestational age

| NO | author | OR | OR_LL | OR_UL | Ln or | SE |
| --- | --- | --- | --- | --- | --- | --- |
| 1 | Jong Ki Jung 2024 | 0.98928787 | 0.970044821 | 1.009654112 | -0.010769918 | 0.01020939 |
| 3 | Ting‑Ting Wang 2019 | 0.952 | 0.674 | 1.346 | -0.049190244 | 0.17644449 |
| 5 | Ying-Yao Chen 2012 | 0.906564779 | 0.871914419 | 0.942592164 | -0.09809279 | 0.019883272 |
| 10 | YANG JUFEN 2018 | 0.935 | 0.711 | 1.286 | -0.06720875 | 0.151178437 |
| 11 | CHEN DAN 2017 | 0.948 | 0.705 | 1.274 | -0.053400777 | 0.150948733 |
| 12 | CAO MENGCHEN 2017 | 0.892 | 0.705 | 1.130 | -0.114289146 | 0.120350793 |

10、small for gestational age

| NO | author | OR | OR_LL | OR_UL | Ln or | SE |
| --- | --- | --- | --- | --- | --- | --- |
| 1 | Jong Ki Jung 2024 | 1.90999122 | 1.354493142 | 2.734189594 | 0.647098645 | 0.179185654 |
| 3 | Ting‑Ting Wang 2019 | 0.283 | 0.026 | 3.045 | -1.262308381 | 1.215091745 |
| 12 | CAO MENGCHEN 2017 | 1.045 | 0.483 | 2.26 | 0.044016885 | 0.393648836 |

11、Early-onset sepsis

| NO | author | OR | OR_LL | OR_UL | Ln or | SE |
| --- | --- | --- | --- | --- | --- | --- |
| 3 | Ting‑Ting Wang 2019 | 3.405 | 1.299 | 8.926 | 1.22524494 | 0.491676946 |
| 9 | CAO ZHAOLAN 2022 | 20.661 | 6.951 | 61.409 | 3.028247865 | 0.555783385 |
| 11 | CHEN DAN 2017 | 3.276 | 1.394 | 7.699 | 1.186623166 | 0.435947229 |
| 14 | Jing-jing Pan 2023 | 6.679 | 3.057 | 14.59 | 1.898968276 | 0.398699571 |
